# Diabetic kidney disease in the elderly: prevalence and clinical correlates

**DOI:** 10.1186/s12877-018-0732-4

**Published:** 2018-02-02

**Authors:** Giuseppina T. Russo, Salvatore De Cosmo, Francesca Viazzi, Antonio Mirijello, Antonio Ceriello, Pietro Guida, Carlo Giorda, Domenico Cucinotta, Roberto Pontremoli, Paola Fioretto, the AMD-Annals Study Group

**Affiliations:** 1 Department of Clinical and Experimental Medicine, University of Messina, Messina, Italy; 2 Department of Medical Sciences, Scientific Institute “Casa Sollievo della Sofferenza,”, San Giovanni Rotondo, (FG) Italy; 3 Department of Internal Medicine, University of Genoa and Policlinico San Martino, Genoa, Italy; 4 Institut d’Investigacions Biomediques August Pii Sunyer (IDIBAPS) and Centro de Investigacion Biomedicaen Red de Diabetes y Enfermedades Metabolicas Asociadas (CIBERDEM), Barcelona, Spain; 5 Department of Cardiovascular and Metabolic Diseases, IRCCS Gruppo Multimedica, Sesto San Giovanni, Italy; 6 Associazione Medici Diabetologi, Rome, Italy; 7 Diabetes and Metabolism Unit, ASL, Turin 5, Chieri (TO), Turin, Italy; 8 Department of Medicine, University of Padua, Padua, Italy; 9 Department of Clinical and experimental Medicine, Policlinico Universitario “G. Martino”, via C. Valeria, 98121 Messina, Italy

**Keywords:** Diabetic kidney disease, Elderly, Type 2 diabetes, Cardiovascular disease

## Abstract

**Background:**

Diabetic kidney disease (DKD) is a major burden in elderly patients with type 2 diabetes (T2DM). Low estimated glomerular filtration rate (eGFR+, < 60 mL/min/1.73 m2) and albuminuria (Alb+) are essential for the diagnosis of DKD, but their association with clinical variables and quality of care may be influenced by ageing.

**Methods:**

Here we investigated the association of clinical variables and quality of care measures with eGFR+ and Alb+ in 157,595 T2DM individuals participating to the Italian Association of Clinical Diabetologists (AMD) Annals Initiative, stratified by age.

**Results:**

The prevalence of eGFR+ and Alb+ increased with ageing, although this increment was more pronounced for low eGFR. Irrespective of age, both the eGFR+ and Alb + groups had the worst risk factors profile when compared to subjects without renal disease, showing a higher prevalence of *out-of target* values of HbA1c, BMI, triglycerides, HDL-C, blood pressure and more complex cardiovascular (CVD) and anti-diabetic therapies, including a larger use of insulin

In all age groups, these associations differed according to the specific renal outcome examined: male sex and smoking were positively associated with Alb+ and negatively with eGFR+; age and anti-hypertensive therapies were more strongly associated with eGFR+, glucose control with Alb+, whereas BMI, and lipid-related variables with both abnormalities. All these associations were attenuated in the older (> 75 years) as compared to the younger groups (< 65 years; 65–75 years), and they were confirmed by multivariate analysis. Notably, Q-score values < 15, indicating a low quality of care, were strongly associated with Alb+ (OR 8.54; *P* < 0.001), but not with eGFR+.

**Conclusions:**

In T2DM patients, the prevalence of both eGFR and Albuminuria increase with age. DKD is associated with poor cardiovascular risk profile and a lower quality of care, although these associations are influenced by the type of renal abnormality and by ageing. These data indicate that clinical surveillance of DKD should not be unerestimated in old T2DM patients.

**Electronic supplementary material:**

The online version of this article (10.1186/s12877-018-0732-4) contains supplementary material, which is available to authorized users.

## Background

Kidney dysfunction is rising worldwide in parallel with population ageing [1], being diagnosed in about 25% of people aged 65–74 years, and in > 50% of those aged > 75 years [2]. Senescence is associated with a progressive decline of estimated glomerular filtration rate (eGFR) of about 1–2 ml/min per year, depending on ethnic, genetic and environmental factors, which may limit renal reserve and make this organ more susceptible to damage by several factors, including type 2 diabetes mellitus (T2DM) [3–6].

T2DM is a progressive disease [7] whose prevalence also increases with age [8], thus exposing elderly patients to an increased risk of long-term diabetic complications, including diabetic kidney disease (DKD).

Low eGFR and albuminuria are the central features for the diagnosis of DKD, and they may be present together in 12% of T2DM subjects or as isolated forms in 35% of them: 24% as albuminuria with preserved eGFR values, and 11% as isolated low eGFR values (< 60 ml/min) [9].

In the large cohort of the Italian Association of Clinical Diabetologists (AMD) Annals Initiative, we have recently demonstrated that low eGFR and albuminuria may have different associations with clinical variables and risk factors for cardiovascular disease (CVD) [9].

However, in spite of the large prevalence of DKD among elderly T2DM patients, it is still unclear whether ageing modifies the associations of low eGFR and albuminuria with CVD risk factors, a particularly relevant issue when considering that DKD is associated with an enormous CVD burden, even at older ages [10, 11].

Furthermore, quality of diabetes care should be carefully considered in elderly patients with DKD, since management of diabetes and its associated CVD risk factors may be different at different ages.

Therefore, here we explored the prevalence of low eGFR and albuminuria and their associations with CVD risk factors and quality of care indicators in a large cohort of T2DM patients participating to the AMD Annals Initiative, stratified according to a wide age-range.

## Methods

In the present report, we analyzed the data set of electronic medical records, collected between 1 January and 31 December 2011, from 157,595 patients with T2DM followed-up at 207 diabetes centers included in the Italian Association of Clinical Diabetologists (AMD) Annals Initiative. Details on database and data collection have been reported elsewhere [12–14]. Data were collected and centrally analyzed anonymously, and the results were internally approved by the AMD Annals scientific committee. This initiative includes measuring and monitoring of major metabolic laboratory parameters, anthropometric measures, systolic and diastolic blood pressure (BP), as well as the use of specific classes of cardiovascular drugs. In particular, kidney function was assessed by serum creatinine and urinary albumin excretion measurements. eGFR was estimated for each patient using a standardized serum creatinine assay and the CKD-EPI formula. Increased urinary albumin excretion was diagnosed as: i) microalbuminuria if UAE rate was > 20 and ≤ 200 μg/min, or if urinary albumin-to-creatinine ratio (ACR) was > 2.5 mg/mmol in men and > 3.5 mg/mmol in women and ≤30 mg/mmol in both genders, or if urinary albumin concentration was > 30 and ≤300 mg/l; ii) macroalbuminuria if UAE rate was > 200 μg/min, or if ACR was > 30 mg/mmol in both genders, or if urinary albumin concentration was > 300 mg/l. Albuminuria indicated patients with either micro- or macroalbuminuria.

### Quality of care assessment

Quality of care was assessed through a validate score, the Q score [15, 16], which is calculated on a combination of process and outcome indicators, based on levels and treatment of major CVD risk factors (HbA1c, blood pressure, LDL-cholesterol and albuminuria), assigning the highest score when the desired goals were attained, whereas the lowest score was assigned when the patient was not treated for the specific condition despite elevated values or when the patient showed unsatisfactory values despite the treatment. Overall, Q score ranges between 0 and 40, with a higher score reflecting better quality of care.

### Statistical analysis

Data are given as mean values ± standard deviation (SD); and categorical variables as frequencies and percentages. Logistic mixed regression models were used to analyze predictors of eGFR < 60 mL/min/1.73 m2 or albuminuria. Due to the large sample size, odds ratios (ORs) were displayed with 99.9% confidence interval (CI). Diabetes clinics were fitted as random effect to consider possible differences in data across centers. Multivariate models were fitted with a complete-case analysis by including patients for which all data were observed. No missing data replacement was used. For the high number of missing values, a separate multivariate OR was computed for smoking status. The analyses were made using STATA software, Version 14 (StataCorp, College Station, Texas).

## Results

### Clinical characteristics of T2DM participants according to age

Clinical characteristics of the 157,595 T2DM subjects according to age (< 65 yrs., 65–75 yrs., > 75 yrs) are shown in Table 1. Male gender and smokers, as well as BMI values decreased with age, whereas glucose control was comparable (mean HbA1c 7.2–7.3%) in all age-groups, in spite of the increasing diabetes duration.

**Table 1:** Baseline clinical characteristics by age groups

	All	*< 65 years*	*65–75 years*	*> 75 years*
	*n = 157,595*	*n = 58,238*	*n = 56,682*	*n = 42,675*
Male sex	89,290 (56.7%)	35,941 (61.7%)	32,469 (57.3%)	20,880 (48.9%)
Age (years)	68 ± 11	57 ± 7	70 ± 3	80 ± 4
Known duration of diabetes (years)	11 ± 9	8 ± 7	12 ± 9	15 ± 11
Serum creatinine (mg/dL)	0.98 ± 0.54	0.88 ± 0.43	0.99 ± 0.54	1.10 ± 0.63
eGFR (mL/min/1.73m^2^)	76 ± 21	89 ± 18	74 ± 18	62 ± 19
Retinopathy	22,250 (14.1%)	6998 (12.0%)	8773 (15.5%)	6479 (15.2%)
BMI (Kg/m^2^)	29.6 ± 5.3	30.4 ± 5.8	29.6 ± 5.1	28.4 ± 4.7
HbA1c (%)	7.2 ± 1.3	7.3 ± 1.4	7.2 ± 1.2	7.2 ± 1.2
HbA1c				
7.0–7.4%	26,576 (17.1%)	9002 (15.7%)	10,057 (18.0%)	7517 (17.9%)
7.5–8.5%	32,320 (20.8%)	11,265 (19.7%)	11,473 (20.5%)	9582 (22.8%)
> 8.5%	20,993 (13.5%)	9214 (16.1%)	6401 (11.4%)	5378 (12.8%)
Total cholesterol (mg/dL)	177 ± 39	181 ± 40	175 ± 37	176 ± 38
Triglycerides (mg/dL)	137 ± 90	147 ± 108	133 ± 82	128 ± 69
HDL (mg/dL)	50 ± 14	48 ± 14	50 ± 14	51 ± 15
LDL (mg/dL)	101 ± 33	104 ± 34	99 ± 32	100 ± 33
Systolic BP (mmHg)	137 ± 18	134 ± 18	139 ± 18	140 ± 19
Diastolic BP (mmHg)	78 ± 9	79 ± 9	78 ± 9	76 ± 9
Smokers	14,793 (16.7%)	8865 (25.2%)	4435 (14.1%)	1493 (6.9%)
Cardiovascular therapy				
Lipid-lowering treatment	90,690 (57.5%)	31,402 (53.9%)	35,490 (62.6%)	23,798 (55.8%)
Treatment with statins	83,342 (52.9%)	28,002 (48.1%)	32,928 (58.1%)	22,412 (52.5%)
Treatment with fibrates	4588 (2.9%)	2285 (3.9%)	1503 (2.7%)	800 (1.9%)
Antihypertensive treatment	112,424 (71.3%)	35,069 (60.2%)	43,031 (75.9%)	34,324 (80.4%)
Treatment with ACE-Is/ARBs	95,821 (60.8%)	30,460 (52.3%)	36,938 (65.2%)	28,423 (66.6%)
Aspirin	35,284 (22.4%)	9467 (16.3%)	14,278 (25.2%)	11,539 (27.0%)
Antidiabetic therapy				
Diet	8229 (5.2%)	3149 (5.4%)	3086 (5.4%)	1994 (4.7%)
Oral antidiabetic drugs	100,535 (63.8%)	39,150 (67.2%)	36,379 (64.2%)	25,006 (58.6%)
Oral antidiabetic drugs and insulin	26,634 (16.9%)	9460 (16.2%)	9906 (17.5%)	7268 (17.0%)
Insulin	22,197 (14.1%)	6479 (11.1%)	7311 (12.9%)	8407 (19.7%)
Q Score	30 ± 8	30 ± 8	30 ± 8	29 ± 8
< 15	5935 (3.8%)	2534 (4.4%)	1808 (3.2%)	1593 (3.7%)
15–25	42,165 (26.8%)	15,375 (26.4%)	14,447 (25.5%)	12,343 (28.9%)
> 25	109,495 (69.5%)	40,329 (69.2%)	40,427 (71.3%)	28,739 (67.3%)

Mean ± SD or absolute frequency (percentage). *ACE*-Is = angiotensin converting enzyme-inhibitors, *ARBs* = angiotensin II receptor antagonists, *BMI* = body mass index, *BP* = blood pressure, *eGFR* = estimated glomerular filtration rate, *HbA1c* = glycated hemoglobin, *HDL* = high-density lipoprotein cholesterol, *LDL* = low-density lipoprotein cholesterol. Patients’ missing data: known duration of diabetes in 8435 (5.4%), BMI in 14,918 (9.5%), HbA1c in 2291 (1.5%), total cholesterol in 8127 (5.2%), triglycerides in 10,293 (6.5%), HDL-c in 12,812 (8.1%), LDL-c in 13,495 (8.6%), blood pressure in 16,009 24,106 (15.3%), and smoking status in 69,213 (43.9%)

eGFR values decreased, whereas serum creatinine levels and the frequency of subjects with diabetic retinopathy increased with age. The oldest groups showed a more favorable lipid profile, higher systolic and lower diastolic BP values and lower proportion of smokers.

Age-differences were also noted in hypoglycaemic therapies: diet therapy only and oral hypoglycaemic drugs use progressively decreased with age, while insulin treatment increased. In particular, in the > 75 years old group, 59% of study subjects were treated with oral drugs, 20% with insulin, 17% with a combination therapy and 5% with diet alone.

As for cardiovascular medications, lipid-lowering treatments were more frequently used in the intermediate age-group, fibrates in the youngest subjects, although in a small absolute number, whereas anti-hypertensive and aspirin treatments progressively increased across age-strata.

Quality of care, assessed through the Q-score, showed high values (mean score 30), indicative of an overall good quality of care, with slightly lower scores in the oldest group.

### Clinical characteristics of T2DM participants according to age and the presence of low eGFR and albuminuria

**Fig. 1:**
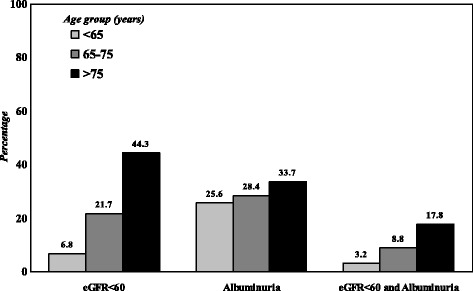
Proportion of patients with eGFR< 60 mL/min/1.73 m2 or albuminuria, by age groups

As shown in Fig. 1, the prevalence of DKD progressively increased with age, and this increment was more evident for low eGFR than for albuminuria (*P* < 0.001 for all comparisons).

Clinical parameters according to age and the presence of low eGFR (Table 2) or albuminuria (Table 3) were also investigated.

**Table 2:** Patients’ characteristics by the presence of low estimated glomerular filtration and by age groups

	*< 65 years*			*65–75 years*			*> 75 years*		
	*eGFR-*	*eGFR+*		*eGFR-*	*eGFR+*		*eGFR-*	*eGFR+*	
	*n = 54,297*	*n = 3941*	*p*	*n = 44,378*	*n = 12,304*	*p*	*n = 23,754*	*n = 18,921*	*p*
Male sex	**33,640 (62.0%)**	**2301 (58.4%)**	**< 0.001**	**25,773 (58.1%)**	**6696 (54.4%)**	**< 0.001**	**12,262 (51.6%)**	**8618 (45.5%)**	**< 0.001**
Age (years)	**56 ± 7**	**60 ± 5**	**< 0.001**	**70 ± 3**	**71 ± 3**	**< 0.001**	**80 ± 4**	**81 ± 4**	**< 0.001**
Duration of diabetes (years)	**8 ± 7**	**11 ± 9**	**< 0.001**	**11 ± 9**	**14 ± 9**	**< 0.001**	**14 ± 10**	**16 ± 11**	**< 0.001**
Albuminuria	**13,031 (24.0%)**	**1855 (47.1%)**	**< 0.001**	**11,126 (25.1%)**	**5000 (40.6%)**	**< 0.001**	**6770 (28.5%)**	**7605 (40.2%)**	**< 0.001**
Retinopathy	**6073 (11.2%)**	**925 (23.5%)**	**< 0.001**	**6208 (14.0%)**	**2565 (20.8%)**	**< 0.001**	**3168 (13.3%)**	**3311 (17.5%)**	**< 0.001**
BMI (Kg/m^2^)	**30.3 ± 5.7**	**31.8 ± 6.2**	**< 0.001**	**29.3 ± 5.0**	**30.5 ± 5.3**	**< 0.001**	**28.0 ± 4.6**	**28.8 ± 4.8**	**< 0.001**
HbA1c (%)	**7.3 ± 1.4**	**7.4 ± 1.5**	**< 0.001**	**7.1 ± 1.2**	**7.3 ± 1.3**	**< 0.001**	**7.2 ± 1.2**	**7.3 ± 1.3**	**< 0.001**
Triglycerides ≥150 mg/dl	**17,380 (33.8%)**	**1916 (52.0%)**	**< 0.001**	**10,902 (26.1%)**	**4792 (42.0%)**	**< 0.001**	**4696 (21.4%)**	**5781 (33.8%)**	**< 0.001**
HDL < 40/< 50 mg/dL (Male/Female)	**19,366 (38.3%)**	**1837 (51.1%)**	**< 0.001**	**12,780 (31.1%)**	**4939 (44.2%)**	**< 0.001**	**6188 (28.6%)**	**6998 (41.7%)**	**< 0.001**
LDL ≥100 mg/dL	**26,449 (52.8%)**	**1593 (45.1%)**	**< 0.001**	**18,723 (45.6%)**	**4820 (43.4%)**	**< 0.001**	10,034 (46.5%)	7676 (45.9%)	0.061
Blood Pressure ≥ 140/85 mmHg	**22,307 (47.6%)**	**1795 (54.0%)**	**< 0.001**	21,453 (56.1%)	5701 (56.0%)	0.130	**11,688 (58.8%)**	**8518 (56.6%)**	**< 0.001**
Smokers	**8406 (25.5%)**	**459 (20.4%)**	**< 0.001**	**3612 (14.5%)**	**823 (12.2%)**	**< 0.001**	**923 (7.7%)**	**570 (6.0%)**	**< 0.001**
Cardiovascular therapy									
Lipid-lowering treatment	**28,831 (53.1%)**	**2571 (65.2%)**	**< 0.001**	**27,130 (61.1%)**	**8360 (67.9%)**	**< 0.001**	**12,802 (53.9%)**	**10,996 (58.1%)**	**< 0.001**
Treatment with statins	**25,854 (47.6%)**	**2148 (54.5%)**	**< 0.001**	**25,456 (57.4%)**	**7472 (60.7%)**	**< 0.001**	**12,224 (51.5%)**	**10,188 (53.8%)**	**< 0.001**
Treatment with fibrates	**1982 (3.7%)**	**303 (7.7%)**	**< 0.001**	**927 (2.1%)**	**576 (4.7%)**	**< 0.001**	**310 (1.3%)**	**490 (2.6%)**	**< 0.001**
Antihypertensive treatment	**31,773 (58.5%)**	**3296 (83.6%)**	**< 0.001**	**32,412 (73.0%)**	**10,619 (86.3%)**	**< 0.001**	**18,098 (76.2%)**	**16,226 (85.8%)**	**< 0.001**
Treatment with ACE-Is/ARBs	**27,592 (50.8%)**	**2868 (72.8%)**	**< 0.001**	**27,773 (62.6%)**	**9165 (74.5%)**	**< 0.001**	**14,992 (63.1%)**	**13,431 (71.0%)**	**< 0.001**
Aspirin	**8470 (15.6%)**	**997 (25.3%)**	**< 0.001**	**10,745 (24.2%)**	**3533 (28.7%)**	**< 0.001**	**6079 (25.6%)**	**5460 (28.9%)**	**< 0.001**
Antidiabetic therapy									
Diet	**3010 (5.5%)**	**139 (3.5%)**	**< 0.001**	**2566 (5.8%)**	**520 (4.2%)**	**< 0.001**	**1262 (5.3%)**	**732 (3.9%)**	**< 0.001**
Oral antidiabetic drugs	**37,223 (68.6%)**	**1927 (48.9%)**	**< 0.001**	**30,103 (67.8%)**	**6276 (51.0%)**	**< 0.001**	**15,565 (65.5%)**	**9441 (49.9%)**	**< 0.001**
Oral drugs and insulin	**8685 (16.0%)**	**775 (19.7%)**	**< 0.001**	**7477 (16.8%)**	**2429 (19.7%)**	**< 0.001**	3878 (16.3%)	3390 (17.9%)	0.001
Insulin	**5379 (9.9%)**	**1100 (27.9%)**	**< 0.001**	**4232 (9.5%)**	**3079 (25.0%)**	**< 0.001**	**3049 (12.8%)**	**5358 (28.3%)**	**< 0.001**
Q Score									
< 15	2345 (4.3%)	189 (4.8%)	0.259	1353 (3.0%)	455 (3.7%)	0.002	858 (3.6%)	735 (3.9%)	0.262
15–25	14,230 (26.2%)	1145 (29.1%)	0.002	**10,953 (24.7%)**	**3494 (28.4%)**	**< 0.001**	**6555 (27.6%)**	**5788 (30.6%)**	**< 0.001**
> 25	**37,722 (69.5%)**	**2607 (66.2%)**	**< 0.001**	**32,072 (72.3%)**	**8355 (67.9%)**	**< 0.001**	**16,341 (68.8%)**	**12,398 (65.5%)**	**< 0.001**

The bold values refers to significant (*p* < 0.001) comparisons between patients with eGFR< 60 mL/min/1.73m^2^ (eGFR+) and those with values ≥ 60 mL/min/1.73m^2^ (eGFR-)

**Table 3:** Patients’ characteristics by the presence of albuminuria and by age groups

	*< 65 years*			*65–75 years*			*> 75 years*		
	*ALB-*	*ALB+*		*ALB-*	*ALB+*		*ALB-*	*ALB+*	
	*n = 43,352*	*n = 14,886*	*p*	*n = 40,556*	*n = 16,126*	*p*	*n = 28,300*	*n = 14,375*	*p*
Male sex	**25,532 (58.9%)**	**10,409 (69.9%)**	**< 0.001**	**21,651 (53.4%)**	**10,818 (67.1%)**	**< 0.001**	**12,721 (45.0%)**	**8159 (56.8%)**	**< 0.001**
Age (years)	**56 ± 7**	**57 ± 7**	**< 0.001**	**70 ± 3**	**70 ± 3**	**< 0.001**	**80 ± 4**	**81 ± 4**	**< 0.001**
Duration of diabetes (years)	**8 ± 7**	**9 ± 8**	**< 0.001**	**12 ± 9**	**13 ± 9**	**< 0.001**	**14 ± 11**	**16 ± 11**	**< 0.001**
eGFR < 60 mL/min/1.73m^2^	**2086 (4.8%)**	**1855 (12.5%)**	**< 0.001**	**7304 (18.0%)**	**5000 (31.0%)**	**< 0.001**	**11,316 (40.0%)**	**7605 (52.9%)**	**< 0.001**
Retinopathy	**4346 (10.0%)**	**2652 (17.8%)**	**< 0.001**	**5357 (13.2%)**	**3416 (21.2%)**	**< 0.001**	**3729 (13.2%)**	**2750 (19.1%)**	**< 0.001**
BMI (Kg/m^2^)	**30.1 ± 5.7**	**31.1 ± 5.9**	**< 0.001**	**29.4 ± 5.1**	**30.0 ± 5.1**	**< 0.001**	**28.3 ± 4.7**	**28.5 ± 4.7**	**< 0.001**
HbA1c (%)	**7.2 ± 1.4**	**7.6 ± 1.6**	**< 0.001**	**7.1 ± 1.2**	**7.4 ± 1.3**	**< 0.001**	**7.1 ± 1.2**	**7.4 ± 1.3**	**< 0.001**
Triglycerides ≥150 mg/dl	**13,144 (32.0%)**	**6152 (43.7%)**	**< 0.001**	**10,438 (27.4%)**	**5256 (34.9%)**	**< 0.001**	**6453 (24.8%)**	**4024 (30.7%)**	**< 0.001**
HDL < 40/< 50 mg/dL (Male/Female)	**15,038 (37.3%)**	**6165 (44.4%)**	**< 0.001**	**12,114 (32.4%)**	**5605 (37.9%)**	**< 0.001**	**8308 (32.5%)**	**4878 (38.0%)**	**< 0.001**
LDL ≥100 mg/dL	**21,368 (53.4%)**	**6674 (49.1%)**	**< 0.001**	**17,237 (46.0%)**	**6306 (42.8%)**	**< 0.001**	**12,048 (47.3%)**	**5662 (44.2%)**	**< 0.001**
Blood Pressure ≥ 140/85 mmHg	**16,778 (45.2%)**	**7324 (56.3%)**	**< 0.001**	**18,392 (53.4%)**	**8762 (62.8%)**	**< 0.001**	**12,690 (55.1%)**	**7516 (63.2%)**	**< 0.001**
Smokers	**6062 (23.4%)**	**2803 (29.9%)**	**< 0.001**	**2863 (12.9%)**	**1572 (16.9%)**	**< 0.001**	**869 (6.1%)**	**624 (8.4%)**	**< 0.001**
Cardiovascular therapy									
Lipid-lowering treatment	**22,567 (52.1%)**	**8835 (59.4%)**	**< 0.001**	**24,821 (61.2%)**	**10,669 (66.2%)**	**< 0.001**	15,504 (54.8%)	8294 (57.7%)	0.072
Treatment with statins	**20,167 (46.5%)**	**7835 (52.6%)**	**< 0.001**	**23,040 (56.8%)**	**9888 (61.3%)**	**< 0.001**	14,616 (51.6%)	7796 (54.2%)	0.217
Treatment with fibrates	**1616 (3.7%)**	**669 (4.5%)**	**< 0.001**	1058 (2.6%)	445 (2.8%)	0.045	508 (1.8%)	292 (2.0%)	0.175
Antihypertensive treatment	**24,528 (56.6%)**	**10,541 (70.8%)**	**< 0.001**	**29,651 (73.1%)**	**13,380 (83.0%)**	**< 0.001**	**22,154 (78.3%)**	**12,170 (84.7%)**	**< 0.001**
Treatment with ACE-Is/ARBs	**21,042 (48.5%)**	**9418 (63.3%)**	**< 0.001**	**25,256 (62.3%)**	**11,682 (72.4%)**	**< 0.001**	**18,282 (64.6%)**	**10,141 (70.5%)**	**< 0.001**
Aspirin	**6536 (15.1%)**	**2931 (19.7%)**	**< 0.001**	**9751 (24.0%)**	**4527 (28.1%)**	**< 0.001**	7468 (26.4%)	4071 (28.3%)	0.010
Antidiabetic therapy									
Diet	**2596 (6.0%)**	**553 (3.7%)**	**< 0.001**	**2445 (6.0%)**	**641 (4.0%)**	**< 0.001**	**1502 (5.3%)**	**492 (3.4%)**	**< 0.001**
Oral antidiabetic drugs	**29,916 (69.0%)**	**9234 (62.0%)**	**< 0.001**	**27,180 (67.0%)**	**9199 (57.0%)**	**< 0.001**	**17,437 (61.6%)**	**7569 (52.7%)**	**< 0.001**
Oral drugs and insulin	**6320 (14.6%)**	**3140 (21.1%)**	**< 0.001**	**6365 (15.7%)**	**3541 (22.0%)**	**< 0.001**	**4416 (15.6%)**	**2852 (19.8%)**	**< 0.001**
Insulin	**4520 (10.4%)**	**1959 (13.2%)**	**< 0.001**	**4566 (11.3%)**	**2745 (17.0%)**	**< 0.001**	**4945 (17.5%)**	**3462 (24.1%)**	**< 0.001**
Q Score									
< 15	**864 (2.0%)**	**1670 (11.2%)**	**< 0.001**	**541 (1.3%)**	**1267 (7.9%)**	**< 0.001**	**449 (1.6%)**	**1144 (8.0%)**	**< 0.001**
15–25	**9847 (22.7%)**	**5528 (37.1%)**	**< 0.001**	**8829 (21.8%)**	**5618 (34.8%)**	**< 0.001**	**6948 (24.6%)**	**5395 (37.5%)**	**< 0.001**
> 25	**32,641 (75.3%)**	**7688 (51.6%)**	**< 0.001**	**31,186 (76.9%)**	**9241 (57.3%)**	**< 0.001**	**20,903 (73.9%)**	**7836 (54.5%)**	**< 0.001**

The bold values refers to significant (*p* < 0.001) comparisons between patients with albuminuria (ALB+) and those with normoalbuminuria (ALB -)

As shown in Table 2, subjects in the eGFR+ (eGFR< 60 ml/min) groups showed a lower prevalence of men, an older age and a longer diabetes duration. Albuminuria and retinopathy were also more prevalent in the eGFR + groups, without differences across age-strata. Similarly, irrespective of age, the percentage of *out-of target* CVD risk factors were significantly higher in the eGFR+ than in the eGFR- groups, with the exceptions of BMI which did not show any difference according to eGFR status in the oldest group, of BP control which was comparable in the 65–75 group, of the percentage of smokers, which was lower in the eGFR+ groups in all age-strata, and LDL-C which was more *at target* in the eGFR- group. Also, glucose control was worst in the eGFR+ groups in all age strata and, overall, poorest in the < 65 years group.

As for concomitant treatments, diet and oral medications decreased whereas insulin use alone or in combination with oral agents was higher in the eGFR+ groups; also the percentage of subjects treated with lipid lowering, anti-hypertensive drugs and aspirin was higher in the eGFR+ groups, irrespectively of age.

The number of subjects with low Q-score (< 15, bad quality of care), was higher in the eGFR+ groups at all ages, whereas Q-score values > 25, indicating a good quality of care, had the opposite trend.

Table 3 shows study parameters according to age and the presence of albuminuria (Alb+). Prevalence of male sex, age and diabetes duration were higher in the Alb+ group, irrespective of the age-categories. Also risk factors profile was worst in the Alb+ groups, which showed higher BMI values, poorer glucose, TGs, HDL-C, BP control and smoking habit; conversely, the Alb+ group had a higher percentage of subjects with *at-target* LDL-C values.

Lipid lowering and anti-hypertensive treatments were more frequent in the Alb+ groups, although the differences were not statistically significant at older ages (> 75 years group). Similarly to the eGFR+ groups, more complex hypoglycemic therapies (insulin alone or in combination) were more frequent in the Alb+ groups at all ages.

The Q score showed consistently worst values in the Alb+ groups at all ages; low Q-score values (< 15) were particularly frequent in younger subjects with than without albuminuria (11.2% vs 2%, respectively).

### Factors associated with low eGFR and albuminuria according to age

Univariate associations of low eGFR and albuminuria with study variables according to age strata are illustrated in Additional file 1: Figure S1.

Both eGFR and albuminuria showed significant associations with study variables, although with a different strength and direction, depending on the examined outcome.

Impaired renal function **(**eGFR< 60 ml/min) was significantly associated with age, diabetes duration, BMI, TG/HDL-C, BP, anti-hypertensive and lipid-lowering treatments, and the strength of these associations was attenuated by ageing. Male sex and smoking habit were negatively associated with low eGFR values. Also, higher BP values (≥ 140/85 mmHg) were positively associated with low eGFR in the < 65 years group and negatively in the older ones.

Albuminuria was positively and strongly associated with male sex, glucose control, TG/HDL-C, BP and medications, and smoking. BP control was positively associated with albuminuria at every age-range. LDL-C was negatively associated with the presence of albuminuria. Also in the case of albuminuria the strength of these associations was generally attenuated in the oldest groups.

These relationships were confirmed at multivariate analysis (Table 4). The model for eGFR includes sex, age, BMI, glucose, lipid and BP control, and albuminuria. The model testing albuminuria as dependent variable also included eGFR. In particular, multivariate analysis confirmed that glucose control was not an independent predictor of low eGFR, and the association with BP control was attenuated in the youngest group (< 65 years). As for smoking habit, it was independently and positively associated with albuminuria (OR 1.60; 95%CI: 1.52–1.68), whereas the opposite was noted for low eGFR (OR 0.79; 95% CI 0.74–0.84).

**Table 4:** Multivariate odds ratios for estimated glomerular filtration rate < 60 mL/min/1.73 m^2^ or albuminuria by age groups

	Overall	*p*	< 65 years	*p*	65–75 years	*p*	> 75 years	*p*
*Model for eGFR < 60 mL/min/1.73 m* ^*2*^								
Male sex	**0.85 (0.82–0.88)**	**< 0.001**	**0.83 (0.76–0.90)**	**< 0.001**	**0.84 (0.80–0.88)**	**< 0.001**	**0.86 (0.82–0.91)**	**< 0.001**
Age (×5 years)	**1.81 (1.79–1.83)**	**< 0.001**	**1.75 (1.67–1.82)**	**< 0.001**	**1.90 (1.82–1.99)**	**< 0.001**	**1.77 (1.72–1.83)**	**< 0.001**
BMI (× 5 Kg/m^2^)	**1.19 (1.17–1.21)**	**< 0.001**	**1.13 (1.09–1.17)**	**< 0.001**	**1.18 (1.15–1.21)**	**< 0.001**	**1.23 (1.20–1.27)**	**< 0.001**
Albuminuria	**2.14 (2.06–2.22)**	**< 0.001**	**2.90 (2.66–3.17)**	**< 0.001**	**2.22 (2.10–2.35)**	**< 0.001**	**1.78 (1.68–1.88)**	**< 0.001**
HbA1c (×1%)	1.00 (0.99–1.02)	0.839	1.00 (0.97–1.03)	0.979	1.01 (0.99–1.03)	0.421	1.00 (0.98–1.02)	0.976
Triglycerides ≥150 mg/dl	**1.69 (1.63–1.76)**	**< 0.001**	**1.85 (1.70–2.02)**	**< 0.001**	**1.73 (1.64–1.83)**	**< 0.001**	**1.58 (1.49–1.67)**	**< 0.001**
HDL < 40/< 50 mg/dL (Male/Female)	**1.41 (1.36–1.46)**	**< 0.001**	**1.34 (1.23–1.46)**	**< 0.001**	**1.39 (1.32–1.47)**	**< 0.001**	**1.48 (1.40–1.56)**	**< 0.001**
LDL ≥100 mg/dL	**0.88 (0.86–0.91)**	**< 0.001**	**0.80 (0.74–0.87)**	**< 0.001**	**0.90 (0.85–0.94)**	**< 0.001**	**0.91 (0.87–0.96)**	**< 0.001**
Blood Pressure ≥ 140/85 mmHg	**0.87 (0.84–0.90)**	**< 0.001**	1.00 (0.92–1.09)	0.969	**0.86 (0.82–0.91)**	**< 0.001**	**0.84 (0.79–0.88)**	**< 0.001**
Smoking^a^	**0.79 (0.74–0.84)**	**< 0.001**	**0.73 (0.64–0.83)**	**< 0.001**	**0.81 (0.73–0.90)**	**< 0.001**	0.82 (0.71–0.94)	0.004
Model for Albuminuria								
Male sex	**2.09 (2.02–2.15)**	**< 0.001**	**1.94 (1.83–2.05)**	**< 0.001**	**2.30 (2.18–2.42)**	**< 0.001**	**2.05 (1.93–2.17)**	**< 0.001**
Age (×5 years)	**1.07 (1.06–1.08)**	**< 0.001**	**1.02 (1.01–1.04)**	**0.011**	**1.08 (1.04–1.13)**	**< 0.001**	**1.16 (1.11–1.20)**	**< 0.001**
BMI (×5 Kg/m^2^)	**1.11 (1.09–1.12)**	**< 0.001**	**1.14 (1.12–1.17)**	**< 0.001**	**1.11 (1.09–1.14)**	**< 0.001**	1.05 (1.02–1.08)	0.003
eGFR< 60 mL/min/1.73 m^2^	**2.19 (2.11–2.27)**	**< 0.001**	**2.99 (2.74–3.27)**	**< 0.001**	**2.27 (2.14–2.40)**	**< 0.001**	**1.82 (1.72–1.93)**	**< 0.001**
HbA1c (×1%)	**1.22 (1.20–1.23)**	**< 0.001**	**1.21 (1.19–1.23)**	**< 0.001**	**1.24 (1.21–1.27)**	**< 0.001**	**1.19 (1.16–1.22)**	**< 0.001**
Triglycerides ≥150 mg/dl	**1.26 (1.22–1.31)**	**< 0.001**	**1.40 (1.33–1.48)**	**< 0.001**	**1.19 (1.13–1.26)**	**< 0.001**	**1.19 (1.11–1.27)**	**< 0.001**
HDL < 40/< 50 mg/dL (Male/Female)	**1.19 (1.15–1.23)**	**< 0.001**	**1.18 (1.11–1.24)**	**< 0.001**	**1.21 (1.14–1.28)**	**< 0.001**	**1.18 (1.11–1.26)**	**< 0.001**
LDL ≥100 mg/dL	**0.92 (0.89–0.95)**	**< 0.001**	**0.89 (0.85–0.94)**	**< 0.001**	0.94 (0.89–0.98)	0.010	0.92 (0.87–0.97)	0.002
Blood Pressure ≥ 140/85 mmHg	**1.47 (1.42–1.51)**	**< 0.001**	**1.49 (1.41–1.57)**	**< 0.001**	**1.45 (1.37–1.52)**	**< 0.001**	**1.47 (1.38–1.56)**	**< 0.001**
Smoking^a^	**1.60 (1.52–1.68)**	**< 0.001**	**1.58 (1.47–1.70)**	**< 0.001**	**1.68 (1.54–1.84)**	**< 0.001**	**1.55 (1.35–1.79)**	**< 0.001**

The bold values refers to significant (*p* < 0.001) associations. Complete case analysis performed including patients for which all data were observed. Overall group included 115,493 patients: 23,973 (20.8%) with eGFR< 60 mL/min/1.73m^2^ and 33,598 (29.1% with albuminuria). Group with < 65 years included 43,945 patients: 2844 (6.5%) with eGFR< 60 mL/min/1.73m^2^ and 11,401 (25.9%) with albuminuria. Group with 65–75 years included 42,248 patients: 8746 (20.7%) with eGFR< 60 mL/min/1.73m^2^ and 12,170 (28.8%) with albuminuria. Group with > 75 years included 29,300 patients: 12,383 (42.26%) with eGFR< 60 mL/min/1.73m^2^ and 10,027 (34.2%) with albuminuria

^a^Models including smoking status was analysed in 67,276 patients (27,431 with < 65 years, 24,249 with 65–75, and 15,596 with > 75 years)

Similarly, a low quality of diabetes care (Q score values < 15 vs > 25) was strongly associated with albuminuria (OR 8.54; *P* < 0.001), whereas this association was not noted for low eGFR values (OR 1.10; *P* = 0.004), irrespective of age groups (Additional file 2: Figure S2).

## Discussion

Diabetes care in elderly patients is challenging because of several epidemiological, clinical and economic issues, which are amplified in presence of chronic complications.

The prevalence of diabetes is high in older subjects, affecting more than 20% of subjects > 65 years [17]. Our data, on a large sample of outpatients with T2DM with a wide age-range, showed that renal complications affect 41.3% of this population, and more than 60% of those aged > 75 years.

These prevalence figures are comparable to those reported both in non-diabetic and diabetic cohorts, although with some disparities imputable to differences in study design, disease duration, and T2DM management.

In non-diabetic cohorts, the prevalence of renal disease parallels the ageing process, affecting up-to 56.1% of subjects aged > 75 years [18–21]. However, the NHANES study underlined how diabetes has a stronger impact on renal function than ageing itself, showing that the increasing prevalence of renal impairment in the US population in the period 2005–2008 was related to the increasing trends of diabetes, while the age distribution of that population did not change during the observation [10].

Available data on DKD prevalence among elderly T2DM cohorts are also varying, with a DKD prevalence of 15.1% reported in older adults in the Republic of Ireland [22], whereas, in theT2DM patients aged > 75 years in the ZODIAC-24 study [23], the prevalence of low eGFR was 42% and that of albuminuria 52%. These data are similar to our findings and those reported in the Renal Insufficiency and Cardiovascular Events (RIACE) Italian Multicenter Study (RIACE) cohort [24].

The overall high prevalence of DKD in elderly T2DM patients is likely to be the result of two opposite trajectories, i.e. the high frequency of T2DM in this age-group, and the decreased mortality rate due to a better control of the disease, which may have contributed to increase patients survival, allowing sufficient time to develop chronic diabetes complications [25–28].

The physiological decline of renal function with age may have also played a role, since senescence is associated with a gradual decline of eGFR [3, 4], an observation that was confirmed by our study, wherein the impairment of eGFR across age-strata was much more pronounced than the occurrence of albuminuria (Fig. 1) .

Similarly, in the UK Prospective Diabetes Study (UKPDS) study, older age was a significant predictor for the onset of low eGFR but not of albuminuria, whose prevalence was comparable to our findings: 24.9% in the UKPDS vs. 25.6% in our study subjects in the corresponding age-range (< 65 years old) [29, 30].

Interestingly, our data also show that not all T2DM patients will eventually develop renal impairment even at very old age; indeed a large group of subjects aged > 75 years (*n* = 16.984, 48.5%) were still normo-albuminuric with preserved eGFR values, thus confirming that DKD in elderly patients is not only consequent to the loss of renal function of the ageing kidney. Furthermore, elderly subjects without DKD (mean age 80 years, diabetes duration 14 years) showed an optimal glucose control (mean HbA1c 7.1%), with lipid and BP values close to recommended targets and no weight loss, indicating the importance of controlling major risk factors in order to prevent renal complications even late in life*.*

CVD risk factors control is another important issue in elderly patients, especially in those with DKD. Thus, the health and economic burden associated with DKD is largely related to the high risk for CVD morbidity and mortality and its evolution to end stage renal disease [31–34], which does not spare the elderly population [10, 11]. In the Atherosclerosis Risk in Communities (ARIC) Study, both eGFR and albuminuria were associated with CVD outcomes, although age and other demographic variables had a modulating effect on these associations [10]. Furthermore, other studies and recent meta-analyses confirmed that the relationship between renal function and CVD mortality is largely consistent across age-categories [35–38].

Accordingly, our data show that both low eGFR and albuminuria are associated with a worst CVD risk factors profile in all the considered age-groups, and this was also evident for parameters such as BMI, triglycerides and smoking habit that progressively decrease at older age.

Our data also confirm that, independently of age, the strength of the associations of CVD risk factors is different when considering low eGFR and albuminuria, as previously demonstrated in our and in other cohorts [9, 24, 30]. These differences were particularly evident for gender, BP and glucose control.

Gender has been reported to have consistent and specific associations with DKD features, with potential implications in terms of CVD risk [39–41]. As expected, in the overall population, female gender was more prevalent at older ages, likely because of the female survival advantage. However, gender differences in DKD were maintained even at older age, with T2DM women being more prevalent in the low eGFR group, and men in the albuminuric group.

This finding is in line with several reports showing gender- differences in renal disease both in non-diabetic [42, 43] and diabetic cohorts [9, 29, 44]. Consistent with our findings of male gender being a risk factor for albumuminuria, in the UKPDS (The United Kingdom Prospective Diabetic Study) male gender was a predictor for the incidence of albuminuria, but not of low eGFR values [29]. Similarly, both in the RIACE study [44] and in previous reports from the AMD Annals Initiative [9], the albuminuric phenotype was more frequent in T2DM men, whereas the low eGFR phenotype was more prevalent in women. Furthermore, male gender seems to be a risk factor for DKD progression towards ESRD [45–49], whereas once on dialysis treatment, mortality risk seems to be higher in the female T2DM population [50–53].

In spite of these epidemiological findings, the pathophysiological bases of these gender differences remain still largely unknown, although it should be kept in mind that formulas commonly used for eGFR calculation are influenced by gender [54, 55].

Worst glucose and BP control were also associated with both low eGFR and albuminuria, irrespective of age and in spite of the overall mean *at- target* values in all age-groups, confirming the undisputed role of intensive control of these risk factors in the prevention of microvascular disease. However, at multivariate analysis, the association with HbA1c values remained significant for albuminuria only; the impact of BP values on the risk of low eGFR was attenuated in the oldest group, suggesting that the network of factors related to eGFR decline in T2DM subjects is more complex.

Notably, although glucose control was overall good in all age-groups (mean HbA1c 7.2%), a larger percentage of subjects in the youngest group showed *out-of-target* HbA1c values (> 8.5%), compared to subjects > 65 years, whereas the higher prevalence of HbA1c between 7.5–8.5% at older age in our cohort could be interpreted in the light of current guidelines that suggest to mitigate glucose targets in the elderly [28, 38].

Age had an influence on these associations, attenuating the relationship between renal features and the examined CVD risk factors. This was particularly evident for BP control and anti-hypertensive treatments, that were positively and strongly associated with low eGFR values in the < 65 years group and progressively less at older ages. Also smoking habit, that was positively associated with albuminuria and negatively with low eGFR was influenced by age.

When evaluating quality of diabetes care, through the Q score [15, 16], we found an overall good quality of care (mean values of 29–30) at different age- and DKD strata, and these data are in line with the findings of other diabetic cohorts in Italy [15, 16]. However, older subjects (> 75 years) and those with albuminuria (Alb+) showed lower Q score values compared to the other groups. Notably, the QUASAR study [15] showed that the risk of developing CVD events was 84% greater in patients with a score of < 15 (incidence rate ratio: 1.84; 95% CI 1.29–2.62) and 17% higher in those with a score between 15 and 25 (incidence rate ratio: 1.17; 95% CI 0.93–1.49), as compared with those with a score of > 25 [15]. Our data extended those observations indicating that, irrespectively of age, low Q-score values are a risk factor also for albuminuria, a widely recognized risk factor for CVD.

Several limitations of the study should be acknowledged. Among these, the potential influence of geriatric conditions, which may affect quality of care and DKD outcomes, was not specifically considered; moreover, we did not take into account that the management of DKD in elderly patients is complicated by the higher exposure to drugs’ side effects, including hypoglycemia, and frequent co-morbidities [28, 34, 56]. Also, we did not explore the potential associations of the available classes of drugs with low eGFR and/or albuminuria phenotypes specifically in the elderly population, although a previous analysis of this cohort showed an high percentages of patients treated with those drugs that should be limited or contraindicated by impaired renal function [57, 58].

## Conclusions

In conclusion, in this representative sample of outpatients with T2DM, DKD prevalence, especially low eGFR, was very high in subjects > 65 years old. Both low eGFR and albuminuria were associated with a worst CVD risk factor profile, although these associations changed according to the specific outcome examined and were generally attenuated at older ages. Quality of diabetes care was overall good even at older age, however, when impaired (Q score < 15), it was associated with a higher risk of albuminuria. These data indicate that DKD in elderly patients is still a high-risk condition that deserves full clinical consideration, in order to implement a targeted treatment and improve its outcomes.

## Additional files

Additional file 1: Figure S1. Odds Ratios with 99.9% confidence interval (CI) for eGFR< 60 mL/min/1.73 m2 (2A) or albuminuria (2B), by age groups. (TIFF 286 kb)

Additional file 2: Figure S2. Odds Ratios with 99.9% confidence interval (CI) of Q Score groups for eGFR< 60 mL/min/1.73 m2 or albuminuria, by age groups. (TIFF 134 kb)

## Abbreviations

ACR: Urinary albumin-to-creatinine ratio
CVD: Cardiovascular disease.
DKD: Diabetic kidney disease
eGFR: Estimated glomerular filtration rate
T2DM: Type 2 diabetes
UAE: Urinary albumin excretion
