# Supplementary figures and images for: Diabetic kidney disease in the elderly: prevalence and clinical correlates
Source: BMC Geriatr. 2018 Feb 2;18:38. doi: 10.1186/s12877-018-0732-4 (PMC5797340; doi:10.1186/s12877-018-0732-4)

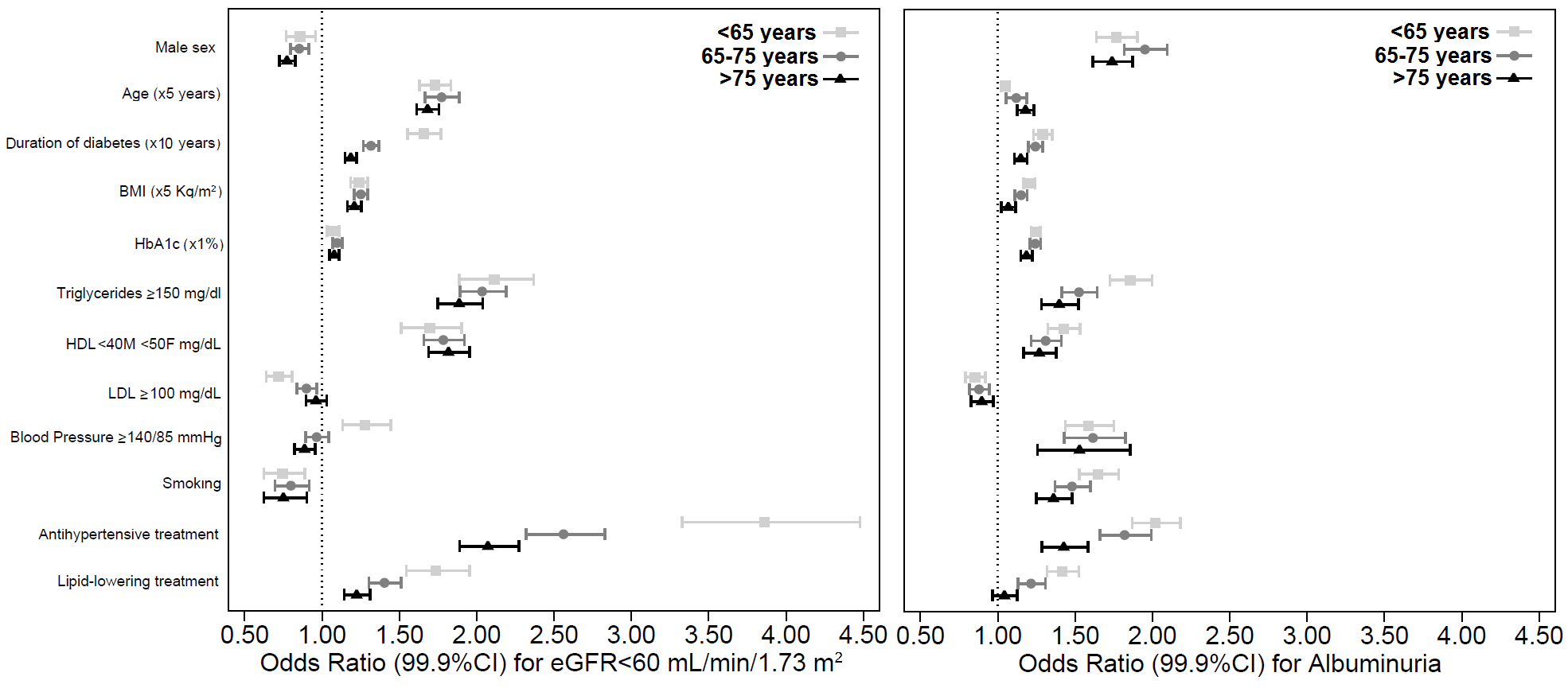

Supplement: Supplementary file 1 — Odds Ratios with 99.9% confidence interval (CI) for eGFR< 60 mL/min/1.73 m2 (2A) or albuminuria (2B), by age groups. (TIFF 286 kb) [file 12877_2018_732_MOESM1_ESM.tif]

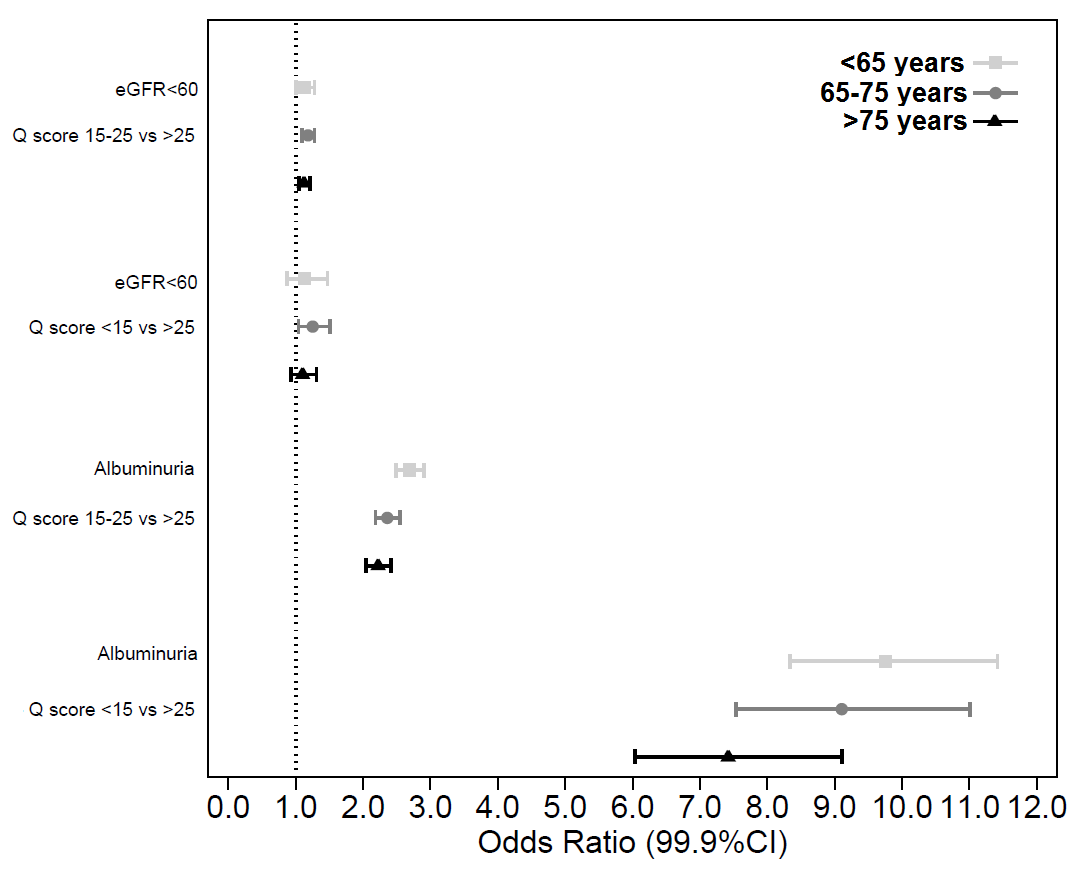

Supplement: Supplementary file 2 — Odds Ratios with 99.9% confidence interval (CI) of Q Score groups for eGFR< 60 mL/min/1.73 m2 or albuminuria, by age groups. (TIFF 134 kb) [file 12877_2018_732_MOESM2_ESM.tif]
